# Secretor Status of ABO Antigens in Saliva of a Defined Group of Iranian Patients with Pemphigus Vulgaris: A Case-Control Study

**DOI:** 10.1155/2020/2950856

**Published:** 2020-07-28

**Authors:** Sedigheh Bakhtiari, Zahra Yadegari, Marziyeh Kaviyani, Zahra Namazi, Mahin Bakhshi

**Affiliations:** ^1^Department of Oral Medicine, School of Dentistry, Shahid Beheshti University of Medical Sciences, Tehran, Iran; ^2^Department of Dental Biomaterials, School of Dentistry, Shahid Beheshti University of Medical Sciences, Tehran, Iran; ^3^School of Dentistry, Shahid Beheshti University of Medical Sciences, Tehran, Iran

## Abstract

**Introduction:**

Pemphigus is a chronic inflammatory and autoimmune disease which can cause blisters and mucocutaneous erosions. ABO secretor refers to those who secrete ABO blood group antigens based on their blood type in body fluids such as saliva, sweat, tears, semen, and serum. Previous studies showed that nonsecretor people are more prone to certain autoimmune diseases.

**Aim:**

The aim of this study was to determine the ABO secretor status in the saliva of patients with pemphigus vulgaris.

**Materials and Methods:**

This case-control study was conducted on 35 patients with pemphigus vulgaris and 35 healthy controls. The two groups were matched for age and gender. Pemphigus vulgaris diagnosis was confirmed by histopathology and direct immunofluorescence microscopy. ABO blood grouping was done, and 5 ml of unstimulated saliva was collected to determine secretor status. Secretors were recognized from nonsecretors by the Wiener agglutination inhibition test. Results were extracted by using statistical chi-square and Fisher's exact tests.

**Results:**

16 male and 19 female patients aged 49.43 ± .12.37 years were compared with 16 male and 19 female controls aged 46.43 ± 11.88 years. The most frequent blood group among case and control groups was O (54.3% and 60%, respectively). There was no significant difference in blood groups (*P*=0.73). 90% of the samples were ABO secretors. The patient group included 31 (88.6%) and the control group included 32 (91.4%) ABO secretors; there was no significant difference between the two groups (*P*=1.000).

**Conclusion:**

In this study, we observed that the people with nonsecretor status in comparison with the people with secretor status are not more susceptible to develop pemphigus vulgaris.

## 1. Introduction

Pemphigus vulgaris (PV), as the most common type of pemphigus, is a chronic and life-threatening disease among autoimmune diseases. Its pathophysiology appears to be due to the harmful circulatory effects of autoantibodies, which are directed against desmosomal components, desmoglein (Dsg1 and Dsg3). These proteins are present in the skin and mucous membranes. Binding of these antibodies to desmoglein causes the separation of adjacent keratinocytes and results in acantholysis and blisters. In addition, it has been suggested that the production of blisters in PV increases the secretion of inflammatory mediators, such as muscarinic receptors expressed by keratinocytes, causing abnormalities in the intercellular signals [1–4].

ABO blood group antigens exist in both red blood cells and body fluids such as saliva, sweat, tears, semen, and serum. The term secretor refers to those who secrete ABO blood group antigens based on their blood type in body fluids. People who do not secrete these antigens in their body fluids are called nonsecretors. Secretor gene is inherited in the form of autosomal dominant. Se gene is the dominant form and se gene is the recessive form; thus, people who are Se Se or Se se are secretors and people who are homozygous se se are nonsecretors [5–7].

Antigen H (H substance) is a precursor for ABO blood group antigens. The related gene is inherited as autosomal dominant. The terms secretor and nonsecretor merely refer to the secretion of ABH antigens. Due to the lack of ABO blood group antigens in body fluids, nonsecretor people are more exposed to endogenous and exogenous antigens than secretor people. In nonsecretor people, lower levels of IgA have been reported in both serum and saliva. This may result in weaker specific immune responses at mucosal surfaces in nonsecretor people compared to the secretors. Moreover, lower levels of IgG have been observed in these patients [8]. This can be one of the causes of increased susceptibility to autoimmune vesiculobullous diseases such as PV, in nonsecretor people. Previous studies have been shown that the nonsecretor people are more prone to autoimmune diseases [9, 10], vaginal candidiasis [11], peptic ulcer [12], submucous fibrosis [13], dental caries [14], gingivitis and periodontal diseases [15, 16], and precancerous lesions [17].

Bakhtiari et al. found that there is no significant difference between the patients with lichen planus and healthy subjects, and more than 80% of people in both groups were secretors [18].

PV has pathophysiological mechanisms similar to the other vesiculobullous diseases. There are few studies about the relationship between secretor status in saliva and pemphigus. Many studies were showed that nonsecretor status could be a risk factor for several diseases [9–18], and thus, we aimed to determine the secretor status in the saliva of patients with PV.

## 2. Materials and Methods

### 2.1. Study Design and Settings

This analytical case-control study was conducted on the patients referred to the Dermatology and Pathology Departments of Shohada and Loghman hospitals (Shahid Beheshti University of Medical Sciences, Tehran, Iran) during 2016-2017. All the participants signed the informed consent form. The design of the study was approved ethically by the Ethics Committee of the Shahid Beheshti Dental School before starting the research (code IR.SBMU.RIDS.REC.1394.122).

### 2.2. Participants

A total of 70 people were selected between the ages of 25 and 75, of which 35 were in the patient group with PV and 35 in the healthy control group. Individuals in both groups were matched for age and gender.

People with a history of other autoimmune diseases or other oral lesions except for pemphigus were excluded from the patient group and the control group included healthy subjects that accompanying the patients.

### 2.3. Data Collection

According to the only somewhat similar study, Shahidi-Dadras and Golfeshan, who found a 42% difference for secretor ratio between the patients and healthy people [19], 35 samples were required in each group for diagnosing this difference in a test with a power of 80% (*α* = 0.05).

Demographic information, location of lesions, blood groups, and family history of the disease were extracted from patients' medical records. The definitive diagnosis of PV was done based on a combination of clinical presentation, histopathology, and direct immunofluorescence (DIF) microscopy (as a gold standard) which can identify tissue-bound autoantibodies. DIF microscopy reveals the intercellular binding of IgG and/or C3 within the epithelium.

The laboratory was asked to identify the blood type for those who had not had a blood type identification card.

### 2.4. Laboratory Procedure

Saliva samples were collected by spitting and Navasesh techniques. 5 ml unstimulated saliva was collected in a 15 ml Falcon tube, while the patient was in a sitting position [20]. The samples were transported to a laboratory, and then, each sample was centrifuged for 15 min at 4°C at 4400 g by a centrifuge (Eppendorf, model 5702, Germany). The supernatants were stored at −70°C. Secretors were recognized from nonsecretors by the Wiener agglutination inhibition test (or Wiener test) [7].

**Figure 1:**
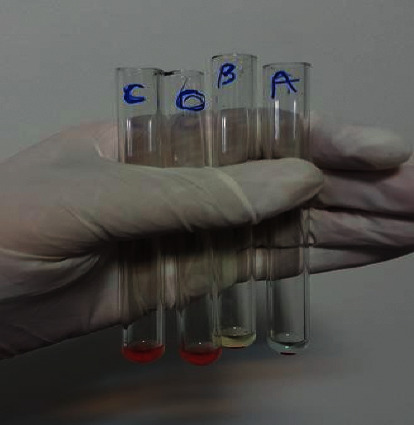
Agglutination is observed in the two tubes at the right side with the signs A and B; it indicates the absence of these antigens in the saliva. In a tube with the O sign, the absence of agglutination indicates the presence of H antigens in saliva. The fourth tube with the C mark is the control tube; absence of agglutination indicates the absence of nonspecific agglutinins in the saliva.

On the same day, samples were thawed at room temperature. Then, the samples were boiled for 10 minutes in a hot water bath (Memmert, Germany). After that, they were centrifuged again for 10 min at 4°C and 3000 g and the supernatants were collected and tested. Each sample was divided into four tubes, and a drop of saliva was added to each of them. One drop of ¼ diluted anti-A (Immunodiagnostika GmbH: A11H5, Germany) was added to the first tube, one drop of ¼ diluted anti-B (Immunodiagnostika GmbH: B-6F9, Germany) was added to the second tube, and one drop of normal saline was added as control to the third and fourth tubes. Tubes were incubated for 20 min at room temperature. Then, one drop of 5% A globule suspension solution was added to the first and the third tubes, and one drop of 5% B globule suspension solution was added to the second and the fourth tubes. Afterward, all tubes were centrifuged for 30 s at 1000 g. Tubes were examined for the presence or absence of hemagglutination. Absence of hemagglutination in tube 1 means neutralization of anti-A secreted by antigen A in saliva resulting in the absence of hemagglutination of blood cell A in this tube. Results obtained for each tube are interpreted in Figure 1.

For samples that showed hemagglutination in both tube 1 and tube 2, one drop of saliva, one drop of ¼ diluted anti-H (Immunodiagnostika GmbH, 10934C11, Germany), and 5% O cell suspension (after incubation at room temperature for 20 min) were added to tube 5. After centrifuge, absence of hemagglutination in this tube means neutralization of anti-H. This was happened by antigen H secreted in saliva that resulted in the absence of hemagglutination of blood cell O in this tube. For this tube, a control tube (tube 6) containing a drop of saliva, a drop of physiology serum, and a drop of 5% O cell suspension was considered.

Absence of hemagglutination in each of the tubes 1, 2, or 5 means that the subject was secretor; only if agglutination was present in all three tubes, the subject was considered nonsecretor. The tubes 3, 4, and 6 were used to control saliva, in some cases.

### 2.5. Statistical Analysis

Mean (SD) and *N* (%) were reported for descriptive statistics. Independent *t*-test was performed to compare patients' mean age between two groups. Chi-square and its exact test were used for comparing the distribution of blood groups and sector status between case and control groups.

The collected data were analyzed using SPSS 21 (IBM Corp., Armonk, NY, USA). The significance level was considered less than 0.05.

## 3. Results

In this case-control study, 16 male and 19 female patients (mean age: 49.43 ± .12.37 years) were compared to 16 males and 19 females in the control group, aged 46.43 ± 11.88 years.

Only 2 of 35 patients (5.7%) reported a positive family history of PV and 33 patients (94.3%) had negative family history.

In terms of lesions' location in the patient group, 10 patients (28.6%) had only oral lesions, 6 patients (17.1%) had only skin lesions, and 19 patients (54.3%) had both oral and skin lesions.

Ninety percent of the samples were secretors. In the patient group, 31 out of 35 patients (88.6%) had secretor status and 4 patients (11.4%) were nonsecretors. In the healthy control group, 32 out of 35 persons (91.4%) had secretor status and 3 persons (8.6%) were nonsecretors. There was no significant difference in secretor status between the patient and control groups (*P*=1.000).

Table 1 shows the results of the studied samples in the patient and control groups.

The most frequent blood type was O in both case and control groups (54.3% and 60%, respectively). There was no significant difference between the two groups, considering their blood groups (*P*=0.73).

## 4. Discussion

ABO blood group system has four main phenotypes: AB, O, B, and A groups. ABO blood group antigens can be present in physiological fluids such as saliva and digestive fluid. The hypothesis that nonsecretor people are more prone to a number of specific bacterial infections or metabolic syndromes and autoimmune disorders led to the evaluation of the relationship between secretor status and the risk of pemphigus which is a life-threatening mucocutaneous disease affecting life quality of the patients [21]. Although the exact mechanism of the relationship between the pathogenesis of PV and secretor blood group antigens has not been described yet, it has been suggested that the nonsecretor individuals have a higher percentage of contamination by pathogens in comparison with secretors, and this may trigger the immune response to different antigens, some of which can mimic self-antigens. Another suggestion is the acceleration of immune system activity as a result of chronic exposure to the various types of antigens [19].

The incidence of this disease in the Iranian population has been reported to be 5 per thousand annually [21]; however, little is known about the relationship between the PV and secretor status.

In many case-control studies, a clear relationship between ABO blood groups and HLA antigens with human disease was revealed. These diseases basically include autoimmune diseases such as PV, type 1 diabetes, multiple sclerosis, rheumatoid arthritis, psoriasis, and celiac disease [22]. The relationship between blood groups and RH with PV has been evaluated in some studies; for example, Shahkar et al. did not find any significant relationship between blood groups and RH with PV among the Iranian population [23]. Likewise, Tirado-Sánchez and Ponce-Olivera have been concluded that there is not any significant relation between ABO blood groups and PV as well [24].

For the first time in 1930, presence or absence of the blood group antigens has been shown in saliva. Schiff showed that these antigens are not present in saliva or other bodily fluids of nonsecretor people who make up about 15% of the world population. AB, B, and A blood groups secrete a large amount of antigens related to their blood groups in their saliva. O group secretes H antigen (FUT1 at1 9q13.3). ABH secretion is controlled by two alleles, Se and se. Se is dominant and se is recessive (or amorphic) [6, 25]. The fucosyltransferase gene family encodes the enzymes that transfer focuses on a large variety of glycans [26]. The secretor gene (FUT2 at 19q13.3) that codes for the activity of the glycosyltransferases is needed to assemble the aspects of both the ABO (FUT1 at1 9q13.3) and Lewis (FUT3 at 19q13.3) blood groups.

Fucosylated oligosaccharides which are present on the cell surface participate in severe biological processes such as cell differentiation, cell movement, inflammation, and adhesions. Saliva and other secretions of ABH secretors contain considerable amounts of carbohydrates in comparison with nonsecretors. This can lead to interference in the above functions [19].

Nurjadi et al. found that nonsecretors with O blood group are at a higher risk for carrying *Staphylococcus aureus* in their throats. However, in secretor people with O blood group, colonization of this bacterium is prevented. The saliva of secretor people contains different groups of oligosaccharides which have different end carbohydrates. Epitopes oligosaccharides are necessary to identify some microorganisms. They are involved in cleaning oral mucosa by trapping and accumulating microorganisms within the secreted substances and preventing damage tissue caused by bacterial enzymes. Nonsecretor people are more likely to be infected by pathogen microorganisms. This can lead to the onset of immune responses to many antigens; some of them imitate the behavior of their antigens [27–29].

Although there is no exact mechanism for the relationship between PV and secretor status, nonsecretor people are more prone to oral mucosal autoimmune responses to their antigens including desmoglein [6].

Shahidi-Dadras and Golfeshan examined secretor status and Lewis phenotypes in patients with PV compared to healthy controls. The total prevalence of nonsecreting phenotypes was significantly higher (*P* < 0.001) in the study group (68%) than in the control group (26%) [19].

To distinguish secretors from nonsecretors, Shahidi examined RBC levels in terms of Lewis antigens [18]. Only in Le (a-b-) people, saliva was examined for the presence of H antigen; they all lacked H antigen and were considered as nonsecretors. However, if these people had a blood group other than O, particularly A1 (blood type is not mentioned), they would have small amounts of H antigen in their secretions [30] and a greater amount of antigens related to their blood group (A or B).

Shahidi-Dadras et al. examined the relationship between secretor status and lichen planus; they reported that nonsecretor status was significantly higher (*P* < 0.001) in patients with lichen planus (74%) than the healthy controls (24%) [9].

Examining the relationship between lichen planus and secretor status, Bakhtiari et al. found no significant difference (*P*=0.73) in secretor status between the patient (16.6%) and control groups (20%) [18].

Vidas et al. also examined the relationship between oral premalignant lesions such as lichen planus and secretor status. The results showed no significant difference (*P*=0.05) in secretor status between the case (14.6%) and control groups (12.9%) [30].

According to this study, it seems that secretor status cannot be considered as a risk factor or preventive factor for autoimmune response to desmoglein levels in the skin and mucosa. Our goal in this study was to determine the ABO secretor status in the saliva of patients with PV; however, some limitations such as few similar studies which directly evaluate the secretory status of blood group antigens in patients with PV, the inadequacy of available samples, and the cost required to perform and advance the work were still encountered.

## 5. Conclusion

People with nonsecretor status may have not more susceptibility than the people with secretor status to develop PV. Although secretion status may contribute to prevent developing some of the diseases, studies have had conflicting results in this regard.

## Figures and Tables

**Table 1:** The results of the studied samples in the patient and control groups.

		Case	Control	*P* value

Gender	Male	16 (45.7%)	16 (45.7%)	1
	Female	19 (54.3%)	19 (54.3%)	
Age		49.49 (12.37)	46.43 (11.88)	0.29

Blood group	A+	7 (20%)	7 (20%)	
	A−	3 (8.6%)	0 (0%)	
	B+	2 (5.7%)	3 (8.6%)	0.73
	B−	3 (8.6%)	2 (5.7%)	
	AB+	1 (2.9%)	2 (5.7%)	
	O+	17 (48.6%)	18 (51.4%)	
	O−	2 (5.7%)	3 (8.6%)	

Secretor status	Secretor	31 (88.6%)	32 (91.4%)	1
	Nonsecretor	4 (11.4%)	3 (8.6%)	

Values are reported as *n* (%) or mean (SD).

## Data Availability

The data used to support the findings of this study are available from the corresponding author upon request.
